# The Role of Fractalkine in Diabetic Retinopathy: Pathophysiology and Clinical Implications

**DOI:** 10.3390/ijms26010378

**Published:** 2025-01-04

**Authors:** Cheng-Yung Lee, Chang-Hao Yang

**Affiliations:** 1 Department of Ophthalmology, National Taiwan University Hospital Hsin-Chu Hospital, No. 25, Ln. 442, Sec. 1, Jingguo Rd., North Dist., Hsinchu City 300195, Taiwan; 2 Department of Ophthalmology, National Taiwan University Hospital, No. 7, Chung Shan S. Rd. (Zhongshan S. Rd.), Zhongzheng Dist., Taipei City 100225, Taiwan; 3 Graduate Institute of Clinical Medicine, College of Medicine, National Taiwan University, No. 7, Chung Shan S. Rd. (Zhongshan S. Rd.), Zhongzheng Dist., Taipei City 100225, Taiwan; 4 Department of Ophthalmology, College of Medicine, National Taiwan University, No. 1 Jen-Ai Road Section 1, Taipei City 10051, Taiwan

**Keywords:** fractalkine, transmembrane fractalkine, soluble fractalkine, fractalkine receptor, CX3CL1-CX3CR1 signaling, inflammation, chemokine, diabetic retinopathy, microglia

## Abstract

Diabetic retinopathy (DR) is a complication of diabetes, characterized by progressive microvascular dysfunction that can result in vision loss. Chronic hyperglycemia drives oxidative stress, endothelial dysfunction, and inflammation, leading to retinal damage and complications such as neovascularization. Current treatments, including anti-VEGF agents, have limitations, necessitating the exploration of alternative therapeutic strategies. Fractalkine (CX3CL1), a chemokine with dual roles as a membrane-bound adhesion molecule and a soluble chemoattractant, has emerged as a potential therapeutic target. Its receptor, CX3CR1, is expressed on immune cells and mediates processes such as immune cell recruitment and microglial activation through intracellular signaling pathways. In DR, soluble fractalkine plays critical roles in retinal inflammation, angiogenesis, and neuroprotection, balancing tissue damage and repair. In DR, elevated fractalkine levels are associated with retinal inflammation and endothelial dysfunction. Experimental studies suggest that fractalkine deficiency exacerbates the severity of diabetic retinopathy (DR), whereas exogenous fractalkine appears to reduce inflammation, oxidative stress, and neuronal damage. However, its role in pathological angiogenesis within DR remains unclear and warrants further investigation. Preclinical evidence indicates that fractalkine may hold therapeutic potential, particularly in mitigating tissue injury and inflammation associated with early-stage DR.

## 1. Introduction

Diabetic retinopathy (DR) is an ocular complication characterized by the gradual onset of microvascular dysfunction associated with diabetes mellitus. This condition leads to a spectrum of clinical manifestations, ranging from mild visual disturbances to severe vision loss and blindness [1,2]. Globally, DR remains a leading cause of blindness, profoundly affecting patients’ quality of life and placing a significant socio-economic burden on healthcare systems [3,4,5]. The pathophysiology of DR is primarily driven by chronic hyperglycemia, which causes structural and functional damage to retinal blood vessels. Key pathological changes include basement membrane thickening, pericyte loss, capillary nonperfusion, and increased vascular permeability. These alterations compromise retinal perfusion and integrity, setting the stage for disease progression [6,7,8]. In advanced stages, neovascularization, a hallmark of proliferative diabetic retinopathy (PDR), emerges and significantly increases the risk of severe complications such as retinal detachment, macular edema, and irreversible vision loss. The substantial morbidity associated with untreated DR highlights the critical importance of early detection and timely intervention to prevent or delay disease progression [9,10,11].

Chronic hyperglycemia in DR triggers a cascade of pathological processes, including oxidative stress, endothelial dysfunction, and inflammation within the retinal microvasculature [12,13]. Among these, inflammation is a critical driver of disease progression, mediated by the activation of immune cells such as monocytes, macrophages, and microglia [14,15]. These immune responses lead to the release of pro-inflammatory cytokines, including tumor necrosis factor-alpha (TNF-α), interleukin-1β (IL-1β), and vascular endothelial growth factor (VEGF), which work together to exacerbate retinal vascular damage and undermine retinal integrity [16,17]. While advancements in understanding the molecular mechanisms of DR have led to effective therapies such as anti-VEGF agents and corticosteroids [18,19], these treatments have limitations. They do not fully prevent capillary dropout or halt underlying disease progression in a substantial subset of patients [20]. This therapeutic gap highlights an urgent need for the development of novel and more efficacious treatment strategies to address the multifaceted pathophysiology of DR.

Among potential therapeutic targets, fractalkine (CX3CL1) has emerged as a crucial mediator of inflammation and immune responses within the retina. Cellular and animal studies have highlighted its potential as a promising candidate for developing innovative treatment strategies in DR [21,22]. Fractalkine is unique in its dual functionality, acting as both a membrane-bound adhesion molecule and a soluble chemokine. In the retina, retinal neurons, particularly retinal ganglion cells, are the primary source of soluble fractalkine, while microglia are the exclusive cell type expressing its receptor, CX3CR1 [23,24]. The interaction between retinal neurons and microglia, facilitated through fractalkine signaling, plays a pivotal role in modulating the pathophysiology of various neurodegenerative and retinal diseases, including DR [25,26,27,28]. Notably, elevated vitreous fractalkine levels were observed in ischemia–reperfusion ocular models. Furthermore, intravitreal injection of fractalkine-neutralizing antibodies was shown to reduce retinal angiogenesis in the oxygen-induced retinopathy animal model, suggesting its involvement in pathological neovascularization [29]. These findings position fractalkine as a compelling target for further exploration in the context of DR treatment.

Recent research underscores the pivotal role of fractalkine in the pathogenesis of DR and highlights its potential as a novel therapeutic target. These findings emphasize the need for a comprehensive evaluation of existing evidence to better understand its clinical implications. In this review, we provide a concise, narrative review of current insights into fractalkine and its receptor, CX3CR1, with a focus on their involvement in critical pathological processes underlying DR. Specifically, we explore their roles in driving retinal inflammation, promoting microglial activation, contributing to endothelial dysfunction, and facilitating pathological angiogenesis.

## 2. Structure and Function of Fractalkine and Its Receptor

### Molecular Structure

Chemokines are a class of small signaling proteins secreted by various cells to recruit immune cells to sites of inflammation [30]. Functionally, chemokines are broadly categorized into two groups: inflammatory chemokines, which are primarily responsible for the recruitment of leukocytes during inflammatory responses [31], and homeostatic chemokines, which play essential roles in the physiological organization and maintenance of secondary lymphoid tissues [32]. Structurally, chemokines are further classified into four subfamilies, C, CC, CXC, and CX3C, based on the arrangement of conserved cysteine residues and the number of amino acids separating the first two cysteines at the N-terminus [33]. This structural and functional diversity enables chemokines to mediate a wide range of immune and physiological processes.

**Figure 1:**
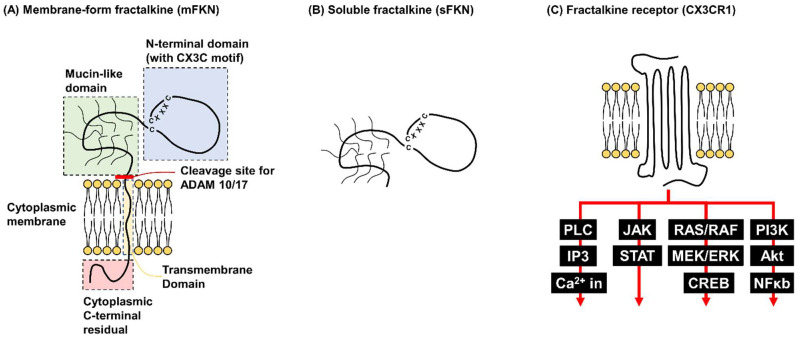
An illustration of membrane-bound fractalkine, soluble fractalkine, and the fractalkine receptor (CX3CR1). (**A**) Membrane-bound fractalkine (mFKN) is a membrane protein consisting of 373 amino acids, structured into four distinct domains: N-terminal, mucin-like, transmembrane, and cytoplasmic domains. It is primarily expressed on vascular endothelial cells and certain immune cells, playing a critical role in recruiting immune cells to inflamed tissues. (**B**) Soluble fractalkine (sFKN) is generated by the cleavage of mFKN via the enzymes ADAM10 and ADAM17. It consists of the N-terminal and mucin-like domains. sFKN is somehow more relevant to diabetic retinopathy pathophysiology than mFKN. (**C**) The fractalkine receptor (CX3CR1) is a G-protein-coupled receptor (GPCR) characterized by seven transmembrane alpha helices. Its activation triggers downstream signaling pathways typical of GPCRs, including PLC/IP3, JAK/STAT, RAS/RAF/MEK, and PI3K/Akt, mediating immune responses, inflammation, and angiogenesis.

Fractalkine, also known as CX3CL1, is a member of the CX3C chemokine family (Figure 1). Within cells, it is initially synthesized as a membrane-bound protein consisting of four distinct domains and a total of 373 amino acids. The extracellular chemokine domain, located at the N-terminus, is composed of 76 amino acids and is connected to a heavily glycosylated mucin-like stalk spanning amino acids 77 to 317 (Figure 1A) [34]. This mucin-like stalk links the chemokine domain to the transmembrane alpha-helix domain (amino acids 318–336), which anchors the protein to the cell membrane, and the cytoplasmic tail (amino acids 337–373) at the C-terminus [35]. The soluble form of fractalkine, which comprises the chemokine domain and part of the mucin-like stalk, is generated through proteolytic cleavage of the membrane-bound protein. This cleavage is mediated by two disintegrin and metalloproteases, ADAM17 and ADAM10, which facilitate the release of soluble fractalkine into the extracellular environment [36,37,38,39].

Similarly to other chemokine receptors, the receptor for fractalkine, CX3CR1, is a G-protein-coupled receptor (GPCR) characterized by its seven transmembrane alpha-helices. Upon ligand binding, CX3CR1 interacts with an associated G protein, which activates intracellular signal transduction pathways to mediate various cellular responses [40].

## 3. General Functional Roles of Fractalkine

Fractalkine exists in two forms, soluble and membrane-bound, enabling it to perform diverse roles in immune regulation [41,42]. Both forms are integral to inflammatory processes, whether physiological or pathological [43,44]. The soluble form functions primarily as a chemoattractant, guiding immune cells such as B cells, T cells, natural killer (NK) cells, and monocytes to sites of inflammation [41,45,46,47]. By recruiting these immune cells, it facilitates immune responses and promotes tissue repair [42,48]. In contrast, membrane-bound fractalkine mediates integrin-independent leukocyte adhesion, acting as a molecular bridge between circulating immune cells and target tissues [49].

This membrane-bound form is expressed by a wide range of cell types [50], including macrophages [51], fibroblasts, activated endothelial cells [50], dendritic cells [52], and neurons [53]. At sites of inflammation, its expression on vascular endothelial cells is upregulated by pro-inflammatory stimuli such as interleukin-1 (IL-1) [45], lipopolysaccharide [54], interferon-gamma (IFN-γ) [54,55], and TNF-alpha [45]. This enhanced expression promotes leukocyte adhesion and facilitates immune cell extravasation into inflamed tissues [45,56]. Furthermore, the co-expression of fractalkine with adhesion molecules such as ICAM-1 and VCAM-1 synergistically strengthens leukocyte adhesion at inflammation sites [57,58]. On the endothelial surface, fractalkine also recruits cytotoxic immune cells, including cytotoxic T lymphocytes and NK cells. These cytotoxic cells can induce local endothelial cell lysis thereby enabling further immune cell migration and amplifying the inflammatory response [59].

As previously noted, the fractalkine receptor, CX3CR1, is a GPCR predominantly expressed on monocytes, T cells, microglia, and natural killer (NK) cells [51,60]. CX3CR1 is integral to mediating fractalkine’s diverse effects, including immune cell recruitment, adhesion, and activation [61]. Upon binding to fractalkine, CX3CR1 undergoes a conformational change characteristic of GPCRs. This structural shift enables its coupling with heterotrimeric G proteins, facilitating the transmission of extracellular signals into the cytoplasm and activating multiple intracellular signaling cascades [62]. Through its GPCR functionality, CX3CR1 engages key downstream signaling pathways, including the phospholipase C (PLC), MAPK/ERK, and PI3K/Akt pathways, which regulate processes such as cell migration, survival, and activation (Figure 1C) [51,63,64]. These pathways highlight CX3CR1’s important role in regulating the immune response and suggest its potential as a therapeutic target in inflammatory and immune-mediated diseases.

Fractalkine regulates the activation state of immune cells, particularly microglia in the central nervous system (CNS) and retina [65]. Pathologically, fractalkine interacts with its receptor, CX3CR1, to modulate microglial activation. This interaction induces chemotaxis, increases intracellular calcium levels, and stimulates the production of pro-inflammatory cytokines and reactive oxygen species (ROS), contributing to tissue damage [63,65]. However, fractalkine also performs essential physiological functions. Neurons express both membrane-bound and soluble forms of fractalkine, which interact with CX3CR1 on microglia to support neuronal and microglial communication [63,65].

During CNS development, fractalkine recruits microglia to developing synapses, where microglia release trophic factors that promote neuronal survival, modulate axon maturation, and facilitate the integration of new neurons [66,67,68]. In cases of neuronal damage, CX3CL1-CX3CR1 signaling activates protective mechanisms in microglia, such as the release of milk fat globule-epidermal growth factor-factor 8 (MFG-E8) to enhance debris clearance and the activation of heme oxygenase-1 (HO-1), which exerts antioxidative effects. These processes highlight the dual role of microglia as contributors to both tissue damage and neuroprotection within neural tissues [69].

Fractalkine mediates vascular pathology during inflammation and angiogenesis [29,56]. In response to inflammatory cytokines such as tumor necrosis, TNF-α and IFN-γ, endothelial cells, upregulate the expression of membrane-bound fractalkine [70]. This expression facilitates the recruitment of CX3CR1-expressing immune cells, including cytotoxic T lymphocytes, natural killer (NK) cells, and macrophages, to the vasculature surrounding inflamed tissues [71]. Membrane-bound fractalkine enables these immune cells to adhere directly to endothelial cells through integrin-independent and selectin-independent mechanisms [72,73]. Furthermore, CX3CR1 activation enhances integrin affinity, stabilizes immune cell adhesion, and promotes integrin-dependent interactions thereby reinforcing immune cell adherence to the endothelium and supporting their extravasation into inflamed tissues [74,75].

## 4. Chemokines and Retinal Cell Interactions in Diabetic Retinopathy

Originating from vascular pathology, DR involves complex interactions among neuronal, vascular, immune, and glial components within the retinal tissue [76,77,78]. The persistent hyperglycemic state induces oxidative stress and inflammation, ultimately disrupting the physiological homeostasis of the retinal tissue [79]. In DR, a range of cytokines and chemokines orchestrate immune responses, facilitating cellular crosstalk and perpetuating chronic inflammation [77]. A network meta-analysis conducted by Pan et al. highlighted CCL2, CCL8, CXCL8, and CXCL10 as chemokines highly associated with DR and diabetic macular edema, with CCL2 and CXCL10 identified as playing critical roles in the pathogenesis of the disease [80].

Microglia, the resident immune cells in retinal tissue, are predominantly activated by ROS generated during chronic hyperglycemia [81]. Once activated, microglia secrete various pro-inflammatory cytokines and chemokines, amplifying the inflammatory response [81]. Among these, CCL2, also called MCP-1, recruits monocytes into the retinal tissue, where they differentiate into macrophages [82]. Under hyperglycemic conditions, macrophages exacerbate inflammation by secreting VEGF and TNF-α, which collectively increase local capillary permeability and drive neovascularization [77]. Additionally, the presence of CXCL8 recruits neutrophil to retinal vessels [83,84]. The adherence of neutrophil to the vascular endothelium leads to leukostasis, capillary obstruction, and subsequent retinal ischemia and tissue damage [85].

In response to chemokines in DR, endothelial cells upregulate the expression of surface adhesion molecules, such as ICAM-1 and VCAM-1, promoting leukocyte adhesion to retinal capillaries and extravasation [86]. This exacerbates capillary leakage and leukostasis, further contributing to retinal vascular dysfunction. Concurrently, chemokines and oxidative stress at retinal capillaries facilitate pericyte loss, driving hallmark pathologies of DR, including capillary leakage, dropout, and microaneurysm formation.

CCL2 and CXCL8 were specifically implicated in the breakdown of the inner blood–retinal barrier [87,88]. CCL2 activates PKC-mediated pathways in vascular endothelial cells, leading to the disruption of tight junction proteins, such as occludin and ZO-1 [89]. Meanwhile, CXCL8 induces VE-cadherin internalization on the vascular endothelium, further compromising barrier integrity [88].

Fractalkine is a unique chemokine, characterized by its dual existence as both membrane-bound and soluble forms. As discussed below, current evidence indicates that, unlike the predominantly harmful effects of chemokines such as CCL2 and CXCL8, fractalkine exerts a more beneficial role by modulating microglial activity. These distinctive characteristics imply its therapeutic potential and make it a particularly compelling subject for further exploration.

## 5. Fractalkine in Diabetic Retinopathy

Fractalkine (CX3CL1) plays a complex and diverse role in the pathophysiology of DR (Table 1) [90]. Elevated levels of soluble fractalkine were detected in the vitreous of patients with DR, highlighting its involvement in the disease [29]. Both chronic inflammation and vascular endothelial dysfunction are critical contributors to the progression of DR [91]. To date, several preclinical or clinical observational studies have explored the roles of fractalkine in DR (Table 2).

In a study using diabetic mice, CD11b+ monocytes demonstrated increased CX3CR1 expression, which was associated with enhanced retinal capillary leukostasis, a hallmark of microvascular inflammation [92]. Additionally, fractalkine expression was found to vary among endothelial cells of different origins. In response to TNF-α, fractalkine expression increased in arterial and capillary endothelium, whereas venous and lymphatic endothelial expression remained unchanged, suggesting a selective endothelial response to inflammatory stimuli [93].

Endothelial cells from different types of blood vessels exhibit varied responses to fractalkine, a phenomenon that may be partially attributed to differences in transcriptional regulation [93,94]. Greene et al. demonstrated that retinal endothelial cells behave distinctly compared to vascular endothelial cells from other regions of the body [95]. While TNF-α typically induces CX3CL1 expression in vascular endothelium [70], this effect is absent in retinal endothelial cells [95]. The response of endothelial cells to inflammatory stimuli appears to depend on the heterogeneity of their vascular origins, influencing the expression of pro-inflammatory molecules and the recruitment of leukocytes [93,96]. Greene et al. identified a unique regulatory pattern of CX3CL1 and TNF-α in retinal endothelial cells. This distinctive regulation may partially explain the retina’s differential susceptibility to inflammation in diabetic retinopathy. They further proposed that the lack of CX3CL1 upregulation in human retinal endothelial cells following CD40 ligation and TNF-α stimulation reflects the tightly controlled inflammatory responses in retina, which are likely aimed at minimizing tissue damage in this highly specialized tissue [95].

In the retina, inner retinal neurons initially express membrane-bound fractalkine, which can be cleaved to release its soluble form into the retinal tissue [21,97]. Tissue-level experiments using retinal explants demonstrated that the application of fractalkine induced localized vasoconstriction, but only in vessels that were in direct contact with microglia. This vasoconstrictive effect was abolished in CX3CR1-null retinas, emphasizing the importance of fractalkine-CX3CR1 signaling [22]. Furthermore, fractalkine application increased the expression of angiotensinogen in retinal explants. The vasoconstrictive effect of fractalkine was eliminated when a type 1 angiotensin receptor antagonist was applied, suggesting a role for the renin–angiotensin system in mediating this response [22].

**Figure 2:**
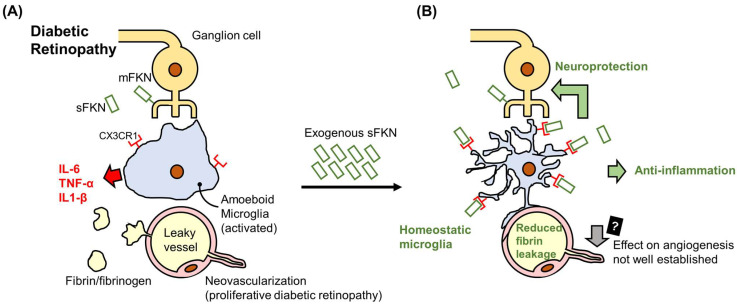
The roles of fractalkine in diabetic retinopathy. (**A**) In the diabetic retina, fractalkine is expressed by injured inner retinal neurons, specifically retinal ganglion cells. Membrane-bound fractalkine is cleaved into its soluble form. Microglia are the only retinal cell type that express the fractalkine receptor, CX3CR1. In diabetic retinopathy (DR), microglia become activated, adopt an amoeboid morphology, and lose their stabilizing influence on retinal capillaries, leading to fibrin or fibrinogen extravasation. Activated microglia also secrete pro-inflammatory mediators, including IL-6, TNF-α, and IL-1β, exacerbating retinal inflammation and neuronal damage. (**B**) Exogenous soluble fractalkine (sFKN) application signals microglia via CX3CR1, inducing a homeostatic state. Microglia morphology is restored, with branched and elongated processes, and the secretion of pro-inflammatory mediators is reduced. Consequently, tissue inflammation and neuronal injury are alleviated. The connection between microglia and capillaries is re-established, decreasing fibrin and fibrinogen leakage. However, the impact of fractalkine on diabetic retinal neovascularization remains poorly understood.

In DR, fractalkine expression by inner retinal neurons and the involvement of microglia play critical roles in modulating neurotoxicity [97,98,99]. Microglia are the sole retinal cell type expressing the fractalkine receptor, CX3CR1 [97]. Fractalkine exerts inhibitory effects on microglial activation, which are essential for maintaining neural homeostasis [25,100,101]. In diabetic mouse models, the absence of CX3CR1 (CX3CR1 knockout) in the retina is associated with heightened microglial activation, neuronal loss, and astrogliosis, along with exacerbated extravasation of fibrin and fibrinogen into retinal tissue (Figure 2A) [24,99,102].

Studies in the Ins2^Akita^ type I diabetic mouse model have demonstrated increased fractalkine expression, emphasizing its regulatory role in the diabetic retina [99]. However, in CX3CR1-deficient Ins2^Akita^ mice (Ins2^Akita^ CX3cr1-/-), neuronal injury and loss are significantly aggravated. These mice exhibit altered microglial morphology, characterized by a transition from highly branched to amoeboid forms with truncated processes, perivascular clustering, and increased proliferation, contributing to elevated neuronal loss (Figure 2A) [99,102,103,104]. In addition, CX3CR1-deficient microglia produce pro-inflammatory mediators, including NOS2, IL-1β, and TNF-α, while anti-inflammatory cytokines such as IL-10 and IL-13 are downregulated. These changes amplify the inflammatory response and oxidative stress, further impairing retinal integrity [105,106]. Astrocytes also play a key role in this inflammatory cascade. In Ins2^Akita^ CX3cr1-/- retinas, astrocytes are a significant source of IL-1β, while CX3CR1-deficient microglia amplify inflammation through additional IL-1β release, contributing to neuronal damage. This inflammatory milieu, characterized by elevated levels of nitric oxide, VEGF, and nitrite, exacerbates retinal dysfunction [99]. Morphological and molecular analyses revealed that these changes are accompanied by reduced ganglion cell counts, lower neuron density, increased microglial activity, and impaired astrocytic responses in 20-week-old Ins2^Akita^ CX3cr1-/- mice. In younger, 10-week-old Ins2^Akita^ CX3cr1-/- mice, similar inflammatory patterns emerge, with the upregulation of IL-1β, NOS2, and TNF-α and the downregulation of IL-10 and IL-13 at the transcript level [99].

Further studies have shown that fractalkine knockout mice exhibit reduced TUJ1+ axon density and diminished SYP+ presynaptic vesicle staining in the optic nerve, revealing the neuroprotective role of fractalkine in preserving retinal neuronal health [21]. The findings suggest that in the diabetic retina, cytokines such as IL-1β produced by microglia and astrocytes, alongside oxidative stress, significantly contribute to neuronal damage. These effects are markedly intensified in the absence of CX3CR1, highlighting the important role of the fractalkine/CX3CR1 axis in mitigating retinal inflammation and neurodegeneration [99]. Given its neuroprotective potential, fractalkine emerges as a promising therapeutic target, particularly in the early stages of diabetic retinopathy [99].

In neural tissues and organs, fractalkine modulates inflammatory and degenerative processes through its interaction with CX3CR1-expressing microglia, a pathway that plays a significant role in several neurodegenerative diseases [65,107,108]. In DR, microglia contribute to early vasculopathy and inflammation [22]. Hallmark features of DR, such as pericyte loss, vasculopathy, and vascular leakage, are closely tied to the activation of microglia in response to serum content leakage into retinal tissue [2,109]. This activation exacerbates local inflammation, further aggravating neuronal damage and perpetuating vascular dysfunction [21]. In the early stages of DR, elevated levels of fractalkine and oxygen in retinal tissue impair vasoconstriction, promoting capillary lumen reduction and closure. The downstream activation of the renin–angiotensin system takes part in microglia-mediated vasoregulation [22]. Additionally, the release of IL-1β during this phase contributes to vascular damage, which is further amplified by activated microglia [99]. An increased association between microglia and retinal capillaries was observed at this stage, reflecting the close interplay between microglial activation and vascular pathology [90].

Studies in a rat model of DR have demonstrated elevated fractalkine levels and increased release of angiotensinogen from microglia as early as four weeks after the onset of diabetes. Interestingly, the elevation of fractalkine was found to reduce the vasoconstrictive effect of angiotensin thereby normalizing the vaso-regulatory function of retinal capillaries [90]. These findings highlight the complex role of fractalkine and microglia in early DR, suggesting that fractalkine may serve both protective and pathological functions depending on the context of its expression and activation.

The administration of exogenous recombinant soluble fractalkine to fractalkine knockout retinas effectively reduced microglial clustering and fibrin or fibrinogen extravasation in murine models of DR [102]. Furthermore, repopulating the retina with CX3CR1-dysfunctional microglia exacerbated DR severity, highlighting the importance of the fractalkine/CX3CR1 signaling pathway in mitigating microglial hyperactivation and excessive inflammation. These findings demonstrate that fractalkine administration can attenuate tissue injury in DR (Figure 2B) [103,110].

The proangiogenic effects of fractalkine/CX3CR1 in inflammatory processes were extensively studied in other tissues [111]. In DR however its proangiogenic role remains inadequately established. Jiang et al. conducted a study that suggested role of fractalkine/CX3CR1 signaling in attenuating neuroinflammation by deactivating microglia in a Sprague Dawley rat model of DR. Using cellular models, including hypoxia-treated microglia that mimic the diabetic retinal microenvironment, they revealed a progressive decline in fractalkine concentration during diabetes progression. This reduction was accompanied by increased microglial activation and elevated levels of inflammatory mediators within diabetic retinal tissue [112]. Under hypoxic conditions, microglia exhibited the upregulated expression of NF-κB, a transcription factor central to inflammatory processes, as well as increased intracellular reactive oxygen species, which are critical drivers of oxidative stress. Additionally, the expression of pro-inflammatory cytokines, including ICAM-1, TNF-α, IL-1β, and IL-6, was significantly elevated, amplifying the inflammatory response [112].

Intravitreal injection of exogenous fractalkine effectively inhibited NF-κB activation while enhancing the Nrf2 pathway, a key regulator of antioxidant defense. The translocation of Nrf2 into the nucleus upregulated the expression of antioxidant-related genes, leading to a reduction in intracellular ROS levels [53,113,114,115]. This attenuation of oxidative stress was accompanied by a decrease in the expression of pro-inflammatory cytokines, including ICAM-1, TNF-α, IL-1β, and IL-6, demonstrating fractalkine’s dual anti-inflammatory and antioxidative effects [112]. Furthermore, the reduced expression of microglial markers such as Iba-1 and TSPO in diabetic retinal tissue provided additional evidence of fractalkine’s efficacy in mitigating microglial overactivation. These changes were notably observed following intravitreal fractalkine administration, further supporting its therapeutic potential in DR [112]. These findings emphasize the protective role of fractalkine in mitigating diabetes-induced retinal damage. This protective effect is achieved through several mechanisms, including the deactivation of microglia, inhibition of the pro-inflammatory NF-κB pathway, and activation of the antioxidant Nrf2 pathway. Together, these actions reduce the production of inflammation-related cytokines and oxidative stress markers thereby limiting retinal inflammation and tissue damage associated with diabetes progression [112].

To explore the roles of fractalkine and microglia in the early stages of diabetic retinopathy (DR), Rodríguez et al. employed recombinant adeno-associated viral vectors (rAAV) carrying plasmids encoding either membrane-bound or soluble fractalkine to transfect retinal tissue, enhancing fractalkine expression prior to diabetes induction [21]. Their study revealed that soluble fractalkine exerted significant protective effects against both vascular and neuronal damage, whereas the membrane-bound form showed minimal impact. Soluble fractalkine notably reduced microglia-associated inflammation, decreased microgliosis, and improved retinal neuronal health. Additionally, reductions in fibrin and fibrinogen leakage from retinal vessels, along with improved visual acuity, were observed in diabetic mouse models treated with soluble fractalkine [21]. Flow cytometry further demonstrated that rAAV–sFKN (soluble fractalkine) treatment reduced the population of reactive microglia, identified by the surface marker Ly6C+, while increasing the population of homeostatic microglia, marked by P2RY12+. Morphological analysis revealed that rAAV–sFKN treatment restored microglial cells in diabetic mice to a ramified state with elongated cellular processes, closely resembling the morphology observed in the non-diabetic controls. Furthermore, treatment with rAAV–sFKN led to a marked reduction in complement components C3 and C1q, which are associated with inflammation and tissue damage [21]. Further investigation into the role of fractalkine in diabetic retinal vessels showed that soluble fractalkine expression in fractalkine knockout diabetic mice reduced retinal vascular tortuosity and preserved tight junction integrity by enhancing connexin-43 and zonula occludens-1 levels [116].

While intravitreal administration of exogenous soluble fractalkine was shown in multiple cellular and animal studies to reduce inflammation and alleviate disease severity in DR, evidence from other disease contexts suggests that fractalkine may also promote angiogenic activity [117,118,119,120]. In a cellular study, You et al. demonstrated that fractalkine treatment enhanced the migration activity of cultured human umbilical vein endothelial cells, suggesting its potential role in angiogenesis [29]. Ahmed et al. further explored the role of fractalkine in the pathophysiology of proliferative diabetic retinopathy (PDR) by analyzing chemokine profiles in the vitreous of PDR patients. Their findings revealed that CXCL16 levels were significantly higher than CX3CL1 levels in vitreous samples, despite a strong positive correlation between VEGF concentrations and the vitreous levels of CXCL16, CX3CL1, ADAM10, and ADAM17 [121]. The study suggested a pro-angiogenic role of fractalkine in PDR. ADAM10 and ADAM17, the key enzymes responsible for cleaving fractalkine from the cell membrane [37,122,123,124], are proposed to play a critical role in modulating its pro-inflammatory and proangiogenic effects within retinal tissue [121]. On the other hand, Rodriguez et al., based on their findings, proposed a potential anti-angiogenic effect of fractalkine due to its ability to enhance vascular integrity and stabilize endothelial tight junctions. However, this hypothesis lacks direct experimental support [116]. Given the absence of direct biochemical evidence, the role of fractalkine in diabetic retinal neovascularization requires further investigation [121].

**Table 1:** Summary of proposed mechanisms of fractalkine/CX3CR1 pathway in diabetic retinopathy.

Affected Pathways or Targets	Effects	Detail	Reference(s)
Inflammation	Anti-inflammation	The activation of the fractalkine/CX3CR1 axis, primarily on microglia, inhibited NF-κB and enhanced Nrf-2, which further decreased intracellular ROS and reduced the levels of pro-inflammatory mediators in retinal tissue, including TNF-α, IL-1β, VEGF, and nitrite.	Cardona et al., 2015 [99], Mendiola et al., 2016 [102], Jiang et al., 2022 [112], Rodriguez et al., 2024 [21]
Retinal ganglion cell	Neuroprotection	The application of fractalkine enhanced the Nrf2 pathway, reduced intracellular ROS levels, and alleviated injury to retinal ganglion cells.	Cardona et al., 2015 [99], Jiang et al., 2022 [112], Rodriguez et al., 2024 [21]
Retinal vessel integrity	Reduction in retinal vascular leakage	The administration of fractalkine reduced retinal vascular leakage and fibrin/fibrinogen extravasation, while also improving vascular endothelial tight junction integrity. At least part of these effects is exerted indirectly through the improvement of the microglial state.	Mendiola et al., 2016 [102], Mills et al., 2021 [22], Rodriguez et al., 2024 [21], Rodriguez et al., 2024 [116]
Microglia	Deactivation of microglia	The fractalkine/CX3CR1 axis signaling transformed activated microglia into a homeostatic state, altering their morphology to exhibit long, branching processes. This signaling also reduced perivascular clustering of microglia. These effects on microglia indirectly alleviated several DR-related tissue injuries.	Kezic et al., 2013 [27], Cardona et al., 2015 [99], Mendiola et al., 2016 [102], Mills et al., 2021 [22] Jiang et al., 2022 [112], Rodriguez et al., 2024 [21] Rodriguez et al., 2024 [116]
Angiogenesis	Lack of definite evidence	No conclusive evidence has yet clarified the relationship between the fractalkine/CX3CR1 axis and diabetic retinal neovascularization.	Abu El-Asrar et al., 2021 [121], Rodriguez et al., 2024 [116]

Abbreviations: ROS = reactive oxidative species; DR = diabetic retinopathy.

**Table 2:** Overview of studies on fractalkine/CX3CR1 pathway in diabetic retinopathy.

Reference	Applied Models/Experimental Subjects	Routes for Exogenous Fractalkine Administration	Key Findings	DR Stage of Interest
Kezic et al., 2013 [27]	Ins2^Akita^ diabetic mice was crossed with CX3CR1-eGFP reporter mice	Not applicable (genetic knockout mouse model applied)	Diabetes disrupts the normal lamellar organization of microglia in the retina. CX3CR1 knockout impaired fractalkine signaling, further disrupted microglial organization and morphology, and increased the accumulation of hyalocytes and macrophages.	Early-stage
Cardona et al., 2015 [99]	Ins2^Akita^ diabetic mice with or without CX3CR1 knockout	Not applicable (genetic knockout mouse model applied)	The knockout of CX3CR1 disrupts fractalkine signal transduction in microglia within the diabetic retina, which, in turn, exacerbates the inflammatory response, neuronal damage, and prolonged microglial activation.	Early-stage
Mendiola et al., 2016 [102]	Ins2^Akita^ diabetic mice with or without CX3CR1 knockout	Intravitreal injection	Knockout of CX3CR1 impairs fractalkine signaling, exacerbates perivascular microglial clustering, increases fibrin/fibrinogen extravasation, and compromises vascular integrity.	Both acute (early) and chronic
Abu El-Asrar et al., 2021 [121]	Analysis of vitreous samples from PDR patients; human cell lines with Muller retina vascular endothelial origins (MIO-M1 and HRMECs)	Not applicable (observational testing was performed on human subjects)	Vitreous fractalkine level was elevated and was found to have a significant positive correlation with the level of VEGF in PDR.	PDR
Mills et al., 2021 [22]	Cx3CR1^GFP/+^ and Cx3CR1^GFP/GFP^ transgenic mice. Diabetes was subsequently induced with STZ	Not applicable (exogenous fractalkine was used in tissue and cellular experiments)	Fractalkine mediates retinal vasoregulation through microglia. Its application induces vasoconstriction in retinal capillaries, an effect abolished by blocking or removing the CX3CR1 receptor. This vasoregulatory process is further linked to the downstream activation of RAS.	Early-stage
Jiang et al., 2022 [112]	STZ-induced diabetic rats; cellular model with glyoxal-treated R28 cells, and hypoxia-treated BV2 cells	Intravitreal injection	Intravitreal administration of fractalkine was shown to deactivate microglia, inhibit NF-κB, and enhance Nrf2 activity, leading to a subsequent reduction in ROS and pro-inflammatory cytokines. These findings suggest a potential neuroprotective effect.	Not specified
Rodriguez et al., 2024 [21]	Fractalkine knockout mice transfected with either mFKN or sFKN using rAAV delivered via intravitreal injection. Diabetes was subsequently induced with STZ	Intravitreal injection of rAAV	sFKN, but not mFKN, dampened microglial activation. Under sFKN expression, fibrin/fibrinogen extravasation was reduced. Examination of mRNA sequencing results revealed neuroprotective, anti-inflammatory, and anti-apoptotic effects. Visual acuity also improved.	Early-stage
Rodriguez et al., 2024 [116]	Fractalkine knockout mice transfected with either mFKN or sFKN using rAAV delivered via intravitreal injection. Diabetes was subsequently induced with STZ	Intravitreal injection of rAAV	Expression of sFKN deactivate microglia, reduce retinal vascular tortuosity, decreased fibrin/fibrinogen extravasation, and maintained integrity of connexin-43 and zonula occludens-1.	Early-stage

Abbreviations: DR = diabetic retinopathy; eGFP = enhanced green fluorescent protein; GFP = green fluorescent protein; PDR = proliferative diabetic retinopathy; STZ = streptozotocin; ROS =reactive oxidative species; mFKN = membrane-bound fractalkine; sFKN = soluble fractalkine; rAAV = recombinant adeno-associated virus; RAS = renin–angiotensin system.

## 6. Administration Routes of Exogenous Fractalkine: Implications from Past Studies and Future Perspectives for Treating Diabetic Retinopathy

All current preclinical studies investigating the therapeutic potential of exogenous fractalkine in DR have employed intravitreal injection in animal models [21,102,112,116]. In CNS research, fractalkine was also delivered via intrathecal injection [125]. It remains uncertain whether serum fractalkine can cross into the vitreous space and achieve therapeutic concentrations in the diabetic retina. On the other hand, intravitreal injection of rAAV encoding soluble fractalkine has shown promising results in mouse models of DR [21,116], suggesting potential as a long-acting therapeutic strategy for humans. However, despite these encouraging preclinical findings, no published clinical trials have yet examined the efficacy of exogenous fractalkine in DR through any administration route. As such, its therapeutic potential in human patients remains an important area for future research.

## 7. Conclusions

The evidence summarized in this review underscores the versatile roles of fractalkine (CX3CL1) and its receptor CX3CR1 in the pathophysiology of DR. Fractalkine signaling plays a pivotal role in regulating retinal inflammation, endothelial function, microglial activation, and neuroprotection. Preclinical studies have highlighted the protective effects of soluble fractalkine, which attenuates inflammation, preserves neuronal health, and mitigates vascular damage. In contrast, the absence or dysregulation of CX3CR1 exacerbates retinal inflammation and neuronal injury, emphasizing the importance of maintaining a delicate balance in fractalkine signaling for retinal homeostasis.

Despite promising preclinical findings, significant challenges remain in translating these insights into clinical practice. Further research is needed to delineate the precise mechanisms governing fractalkine’s dual role in inflammation and angiogenesis, as well as its long-term effects on retinal structure and function. Such efforts are critical to advancing our understanding of fractalkine’s therapeutic potential and developing targeted treatments that could complement existing therapies. Fractalkine-based interventions hold particular promise for managing DR in its early stages, offering a potential avenue to mitigate disease progression and improve outcomes for patients.

## Data Availability

Not applicable.

## References

[1] Pushparani D.S., Varalakshmi J., Roobini K., Hamshapriya P., Livitha A. (2024). Diabetic Retinopathy—A Review. Curr. Diabetes Rev..

[2] Wang W., Lo A.C.Y. (2018). Diabetic Retinopathy: Pathophysiology and Treatments. Int. J. Mol. Sci..

[3] Nentwich M.M., Ulbig M.W. (2015). Diabetic retinopathy—Ocular complications of diabetes mellitus. World J. Diabetes.

[4] Sabanayagam C., Banu R., Chee M.L., Lee R., Wang Y.X., Tan G., Jonas J.B., Lamoureux E.L., Cheng C.Y., Klein B.E.K. (2019). Incidence and progression of diabetic retinopathy: A systematic review. Lancet Diabetes Endocrinol..

[5] Zayed M.G., Karsan W., Peto T., Saravanan P., Virgili G., Preiss D. (2024). Diabetic Retinopathy and Quality of Life: A Systematic Review and Meta-Analysis. JAMA Ophthalmol..

[6] Lechner J., O’Leary O.E., Stitt A.W. (2017). The pathology associated with diabetic retinopathy. Vis. Res..

[7] Trost A., Bruckner D., Rivera F.J., Reitsamer H.A. (2019). Pericytes in the Retina. Adv. Exp. Med. Biol..

[8] Takagi H. (2003). Molecular mechanisms of retinal neovascularization in diabetic retinopathy. Intern. Med..

[9] Newman D.K. (2010). Surgical management of the late complications of proliferative diabetic retinopathy. Eye.

[10] Pandit S., Ho A.C., Yonekawa Y. (2023). Recent advances in the management of proliferative diabetic retinopathy. Curr. Opin. Ophthalmol..

[11] Mohamed Q., Gillies M.C., Wong T.Y. (2007). Management of diabetic retinopathy: A systematic review. JAMA.

[12] Safi S.Z., Qvist R., Kumar S., Batumalaie K., Ismail I.S. (2014). Molecular mechanisms of diabetic retinopathy, general preventive strategies, and novel therapeutic targets. Biomed. Res. Int..

[13] Kang Q., Yang C. (2020). Oxidative stress and diabetic retinopathy: Molecular mechanisms, pathogenetic role and therapeutic implications. Redox. Biol..

[14] Pan W.W., Lin F., Fort P.E. (2021). The innate immune system in diabetic retinopathy. Prog. Retin. Eye Res..

[15] Zhao B., Zhao Y., Sun X. (2024). Mechanism and therapeutic targets of circulating immune cells in diabetic retinopathy. Pharmacol. Res..

[16] Padovani-Claudio D.A., Morales M.S., Smith T.E., Ontko C.D., Namburu N.S., Palmer S.A., Jhala M.G., Ramos C.J., Capozzi M.E., McCollum G.W. (2024). Induction, amplification, and propagation of diabetic retinopathy-associated inflammatory cytokines between human retinal microvascular endothelial and Müller cells and in the mouse retina. Cell. Signal..

[17] Cheung C.M., Vania M., Ang M., Chee S.P., Li J. (2012). Comparison of aqueous humor cytokine and chemokine levels in diabetic patients with and without retinopathy. Mol. Vis..

[18] Cheung N., Wong I.Y., Wong T.Y. (2014). Ocular anti-VEGF therapy for diabetic retinopathy: Overview of clinical efficacy and evolving applications. Diabetes Care.

[19] Massengill M.T., Cubillos S., Sheth N., Sethi A., Lim J.I. (2024). Response of Diabetic Macular Edema to Anti-VEGF Medications Correlates with Improvement in Macular Vessel Architecture Measured with OCT Angiography. Ophthalmol. Sci..

[20] Zhu Z.Y., Meng Y.A., Yan B., Luo J. (2021). Effect of anti-VEGF treatment on nonperfusion areas in ischemic retinopathy. Int. J. Ophthalmol..

[21] Rodriguez D., Church K.A., Pietramale A.N., Cardona S.M., Vanegas D., Rorex C., Leary M.C., Muzzio I.A., Nash K.R., Cardona A.E. (2024). Fractalkine isoforms differentially regulate microglia-mediated inflammation and enhance visual function in the diabetic retina. J. Neuroinflamm..

[22] Mills S.A., Jobling A.I., Dixon M.A., Bui B.V., Vessey K.A., Phipps J.A., Greferath U., Venables G., Wong V.H.Y., Wong C.H.Y. (2021). Fractalkine-induced microglial vasoregulation occurs within the retina and is altered early in diabetic retinopathy. Proc. Natl. Acad. Sci. USA.

[23] Silverman M.D., Zamora D.O., Pan Y., Texeira P.V., Baek S.H., Planck S.R., Rosenbaum J.T. (2003). Constitutive and inflammatory mediator-regulated fractalkine expression in human ocular tissues and cultured cells. Investig. Ophthalmol. Vis. Sci..

[24] Beli E., Dominguez J.M., Hu P., Thinschmidt J.S., Caballero S., Calzi S.L., Luo D., Shanmugam S., Salazar T.E., Duan Y. (2016). CX3CR1 deficiency accelerates the development of retinopathy in a rodent model of type 1 diabetes. J. Mol. Med..

[25] Cho S.H., Sun B., Zhou Y., Kauppinen T.M., Halabisky B., Wes P., Ransohoff R.M., Gan L. (2011). CX3CR1 protein signaling modulates microglial activation and protects against plaque-independent cognitive deficits in a mouse model of Alzheimer disease. J. Biol. Chem..

[26] Staniland A.A., Clark A.K., Wodarski R., Sasso O., Maione F., D’Acquisto F., Malcangio M. (2010). Reduced inflammatory and neuropathic pain and decreased spinal microglial response in fractalkine receptor (CX3CR1) knockout mice. J. Neurochem..

[27] Kezic J.M., Chen X., Rakoczy E.P., McMenamin P.G. (2013). The effects of age and Cx3cr1 deficiency on retinal microglia in the Ins2(Akita) diabetic mouse. Investig. Ophthalmol. Vis. Sci..

[28] Pabon M.M., Bachstetter A.D., Hudson C.E., Gemma C., Bickford P.C. (2011). CX3CL1 reduces neurotoxicity and microglial activation in a rat model of Parkinson’s disease. J. Neuroinflamm..

[29] You J.J., Yang C.H., Huang J.S., Chen M.S., Yang C.M. (2007). Fractalkine, a CX3C chemokine, as a mediator of ocular angiogenesis. Investig. Ophthalmol. Vis. Sci..

[30] Iwamoto T., Okamoto H., Toyama Y., Momohara S. (2008). Molecular aspects of rheumatoid arthritis: Chemokines in the joints of patients. FEBS J..

[31] Turner M.D., Nedjai B., Hurst T., Pennington D.J. (2014). Cytokines and chemokines: At the crossroads of cell signalling and inflammatory disease. Biochim. Biophys. Acta.

[32] Lipp M., Förster R., Schubel A., Burgstahler R., Müller G., Breitfeld D., Kremmer E., Wolf E. (2000). Functional organization of secondary lymphoid organs by homeostatic chemokines. Eur. Cytokine Netw..

[33] Hughes C.E., Nibbs R.J.B. (2018). A guide to chemokines and their receptors. FEBS J..

[34] White G.E., Greaves D.R. (2012). Fractalkine: A survivor’s guide: Chemokines as antiapoptotic mediators. Arter. Thromb. Vasc. Biol..

[35] Apostolakis S., Spandidos D. (2013). Chemokines and atherosclerosis: Focus on the CX3CL1/CX3CR1 pathway. Acta Pharmacol. Sin..

[36] Winter A.N., Subbarayan M.S., Grimmig B., Weesner J.A., Moss L., Peters M., Weeber E., Nash K., Bickford P.C. (2020). Two forms of CX3CL1 display differential activity and rescue cognitive deficits in CX3CL1 knockout mice. J. Neuroinflamm..

[37] Garton K.J., Gough P.J., Blobel C.P., Murphy G., Greaves D.R., Dempsey P.J., Raines E.W. (2001). Tumor necrosis factor-alpha-converting enzyme (ADAM17) mediates the cleavage and shedding of fractalkine (CX3CL1). J. Biol. Chem..

[38] Hundhausen C., Misztela D., Berkhout T.A., Broadway N., Saftig P., Reiss K., Hartmann D., Fahrenholz F., Postina R., Matthews V. (2003). The disintegrin-like metalloproteinase ADAM10 is involved in constitutive cleavage of CX3CL1 (fractalkine) and regulates CX3CL1-mediated cell-cell adhesion. Blood.

[39] Wildenberg M.E., van Helden-Meeuwsen C.G., Drexhage H.A., Versnel M.A. (2008). Altered fractalkine cleavage potentially promotes local inflammation in NOD salivary gland. Arthritis. Res. Ther..

[40] Kim K.W., Vallon-Eberhard A., Zigmond E., Farache J., Shezen E., Shakhar G., Ludwig A., Lira S.A., Jung S. (2011). In vivo structure/function and expression analysis of the CX3C chemokine fractalkine. Blood.

[41] Imai T., Hieshima K., Haskell C., Baba M., Nagira M., Nishimura M., Kakizaki M., Takagi S., Nomiyama H., Schall T.J. (1997). Identification and molecular characterization of fractalkine receptor CX3CR1, which mediates both leukocyte migration and adhesion. Cell.

[42] Vu T.H., Kim C., Truong A.D., Lillehoj H.S., Hong Y.H. (2024). Unveiling the immunomodulatory role of soluble chicken fractalkine: Insights from functional characterization and pathway activation analyses. Dev. Comp. Immunol..

[43] Chen X., Wei Q., Hu Y., Wang C. (2020). Role of Fractalkine in promoting inflammation in sepsis-induced multiple organ dysfunction. Infect. Genet. Evol..

[44] Souza G.R., Talbot J., Lotufo C.M., Cunha F.Q., Cunha T.M., Ferreira S.H. (2013). Fractalkine mediates inflammatory pain through activation of satellite glial cells. Proc. Natl. Acad. Sci. USA.

[45] Bazan J.F., Bacon K.B., Hardiman G., Wang W., Soo K., Rossi D., Greaves D.R., Zlotnik A., Schall T.J. (1997). A new class of membrane-bound chemokine with a CX3C motif. Nature.

[46] Corcione A., Ferretti E., Bertolotto M., Fais F., Raffaghello L., Gregorio A., Tenca C., Ottonello L., Gambini C., Furtado G. (2009). CX3CR1 is expressed by human B lymphocytes and mediates [corrected] CX3CL1 driven chemotaxis of tonsil centrocytes. PLoS ONE.

[47] Park Y., Lee J., Kwak J.Y., Noh K., Yim E., Kim H.K., Kim Y.J., Broxmeyer H.E., Kim J.A. (2018). Fractalkine induces angiogenic potential in CX3CR1-expressing monocytes. J. Leukoc. Biol..

[48] Böttcher J.P., Beyer M., Meissner F., Abdullah Z., Sander J., Höchst B., Eickhoff S., Rieckmann J.C., Russo C., Bauer T. (2015). Functional classification of memory CD8(+) T cells by CX3CR1 expression. Nat. Commun..

[49] Jones B.A., Beamer M., Ahmed S. (2010). Fractalkine/CX3CL1: A potential new target for inflammatory diseases. Mol. Interv..

[50] Abdin A., Daas L., Pattmoller M., Suffo S., Langenbucher A., Seitz B. (2018). Negative impact of dextran in organ culture media for pre-stripped tissue preservation on DMEK (Descemet membrane endothelial keratoplasty) outcome. Graefes. Arch. Clin. Exp. Ophthalmol..

[51] Lee M., Lee Y., Song J., Lee J., Chang S.Y. (2018). Tissue-specific Role of CX(3)CR1 Expressing Immune Cells and Their Relationships with Human Disease. Immune Netw..

[52] Papadopoulos E.J., Sassetti C., Saeki H., Yamada N., Kawamura T., Fitzhugh D.J., Saraf M.A., Schall T., Blauvelt A., Rosen S.D. (1999). Fractalkine, a CX3C chemokine, is expressed by dendritic cells and is up-regulated upon dendritic cell maturation. Eur. J. Immunol..

[53] Luo P., Chu S.F., Zhang Z., Xia C.Y., Chen N.H. (2019). Fractalkine/CX3CR1 is involved in the cross-talk between neuron and glia in neurological diseases. Brain Res. Bull..

[54] Imaizumi T., Yoshida H., Satoh K. (2004). Regulation of CX3CL1/fractalkine expression in endothelial cells. J. Atheroscler. Thromb..

[55] Yoneda O., Imai T., Nishimura M., Miyaji M., Mimori T., Okazaki T., Domae N., Fujimoto H., Minami Y., Kono T. (2003). Membrane-bound form of fractalkine induces IFN-gamma production by NK cells. Eur. J. Immunol..

[56] Umehara H., Bloom E.T., Okazaki T., Nagano Y., Yoshie O., Imai T. (2004). Fractalkine in vascular biology: From basic research to clinical disease. Arterioscler. Thromb. Vasc. Biol..

[57] Umehara H., Bloom E., Okazaki T., Domae N., Imai T. (2001). Fractalkine and vascular injury. Trends Immunol..

[58] Umehara H., Goda S., Imai T., Nagano Y., Minami Y., Tanaka Y., Okazaki T., Bloom E.T., Domae N. (2001). Fractalkine, a CX3C-chemokine, functions predominantly as an adhesion molecule in monocytic cell line THP-1. Immunol. Cell Biol..

[59] Eain H.S., Kawai H., Nakayama M., Oo M.W., Ohara T., Fukuhara Y., Takabatake K., Shan Q., Soe Y., Ono K. (2024). Double-faced CX3CL1 enhances lymphangiogenesis-dependent metastasis in an aggressive subclone of oral squamous cell carcinoma. JCI Insight.

[60] Zhao J., Li Q., Ouyang X., Wang F., Li Q., Xu Z., Ji D., Wu Q., Zhang J., Lu C. (2023). The effect of CX3CL1/ CX3CR1 signal axis on microglia in central nervous system diseases. J. Neurorestor..

[61] Sciumè G., Soriani A., Piccoli M., Frati L., Santoni A., Bernardini G. (2010). CX3CR1/CX3CL1 axis negatively controls glioma cell invasion and is modulated by transforming growth factor-β1. Neuro. Oncol..

[62] Rutti S., Arous C., Schvartz D., Timper K., Sanchez J.C., Dermitzakis E., Donath M.Y., Halban P.A., Bouzakri K. (2014). Fractalkine (CX3CL1), a new factor protecting β-cells against TNFα. Mol. Metab..

[63] Sheridan G.K., Murphy K.J. (2013). Neuron-glia crosstalk in health and disease: Fractalkine and CX3CR1 take centre stage. Open Biol..

[64] Zhuang Z.Y., Kawasaki Y., Tan P.H., Wen Y.R., Huang J., Ji R.R. (2007). Role of the CX3CR1/p38 MAPK pathway in spinal microglia for the development of neuropathic pain following nerve injury-induced cleavage of fractalkine. Brain Behav. Immun..

[65] Harrison J.K., Jiang Y., Chen S., Xia Y., Maciejewski D., McNamara R.K., Streit W.J., Salafranca M.N., Adhikari S., Thompson D.A. (1998). Role for neuronally derived fractalkine in mediating interactions between neurons and CX3CR1-expressing microglia. Proc. Natl. Acad. Sci. USA.

[66] Arnoux I., Audinat E. (2015). Fractalkine Signaling and Microglia Functions in the Developing Brain. Neural Plast..

[67] Nayak D., Roth T.L., McGavern D.B. (2014). Microglia development and function. Annu. Rev. Immunol..

[68] Pérez-Rodríguez D.R., Blanco-Luquin I., Mendioroz M. (2021). The Participation of Microglia in Neurogenesis: A Review. Brain Sci..

[69] Noda M., Doi Y., Liang J., Kawanokuchi J., Sonobe Y., Takeuchi H., Mizuno T., Suzumura A. (2011). Fractalkine attenuates excito-neurotoxicity via microglial clearance of damaged neurons and antioxidant enzyme heme oxygenase-1 expression. J. Biol. Chem..

[70] Matsumiya T., Ota K., Imaizumi T., Yoshida H., Kimura H., Satoh K. (2010). Characterization of synergistic induction of CX3CL1/fractalkine by TNF-alpha and IFN-gamma in vascular endothelial cells: An essential role for TNF-alpha in post-transcriptional regulation of CX3CL1. J. Immunol..

[71] Schulz C., Schäfer A., Stolla M., Kerstan S., Lorenz M., von Brühl M.L., Schiemann M., Bauersachs J., Gloe T., Busch D.H. (2007). Chemokine fractalkine mediates leukocyte recruitment to inflammatory endothelial cells in flowing whole blood: A critical role for P-selectin expressed on activated platelets. Circulation.

[72] Fujita M., Takada Y.K., Takada Y. (2014). The chemokine fractalkine can activate integrins without CX3CR1 through direct binding to a ligand-binding site distinct from the classical RGD-binding site. PLoS ONE.

[73] Nishimura M., Umehara H., Nakayama T., Yoneda O., Hieshima K., Kakizaki M., Dohmae N., Yoshie O., Imai T. (2002). Dual functions of fractalkine/CX3C ligand 1 in trafficking of perforin+/granzyme B+ cytotoxic effector lymphocytes that are defined by CX3CR1 expression. J. Immunol..

[74] Moser B., Loetscher P. (2001). Lymphocyte traffic control by chemokines. Nat. Immunol..

[75] Gerard C., Rollins B.J. (2001). Chemokines and disease. Nat. Immunol..

[76] Zhou J., Chen B. (2023). Retinal Cell Damage in Diabetic Retinopathy. Cells.

[77] Kovoor E., Chauhan S.K., Hajrasouliha A. (2022). Role of inflammatory cells in pathophysiology and management of diabetic retinopathy. Surv. Ophthalmol..

[78] Yang S., Zhang J., Chen L. (2020). The cells involved in the pathological process of diabetic retinopathy. Biomed. Pharmacother..

[79] Brownlee M. (2001). Biochemistry and molecular cell biology of diabetic complications. Nature.

[80] Pan X., Tan X., McDonald J., Kaminga A.C., Chen Y., Dai F., Qiu J., Zhao K., Peng Y. (2024). Chemokines in diabetic eye disease. Diabetol. Metab. Syndr..

[81] Milne R., Brownstein S. (2013). Advanced glycation end products and diabetic retinopathy. Amino Acids.

[82] Tashimo A., Mitamura Y., Nagai S., Nakamura Y., Ohtsuka K., Mizue Y., Nishihira J. (2004). Aqueous levels of macrophage migration inhibitory factor and monocyte chemotactic protein-1 in patients with diabetic retinopathy. Diabet. Med..

[83] de Oliveira S., Reyes-Aldasoro C.C., Candel S., Renshaw S.A., Mulero V., Calado A. (2013). Cxcl8 (IL-8) mediates neutrophil recruitment and behavior in the zebrafish inflammatory response. J. Immunol..

[84] Hou Y., Huttenlocher A. (2024). Advancing chemokine research: The molecular function of CXCL8. J. Clin. Investig..

[85] Omatsu T., Cepinskas G., Clarson C., Patterson E.K., Alharfi I.M., Summers K., Couraud P.O., Romero I.A., Weksler B., Fraser D.D. (2014). CXCL1/CXCL8 (GROα/IL-8) in human diabetic ketoacidosis plasma facilitates leukocyte recruitment to cerebrovascular endothelium in vitro. Am. J. Physiol. Endocrinol. Metab..

[86] Rangasamy S., McGuire P.G., Das A. (2012). Diabetic retinopathy and inflammation: Novel therapeutic targets. Middle East Afr. J. Ophthalmol..

[87] Rangasamy S., McGuire P.G., Nitta C.F., Monickaraj F., Oruganti S.R., Das A. (2014). Chemokine mediated monocyte trafficking into the retina: Role of inflammation in alteration of the blood-retinal barrier in diabetic retinopathy. PLoS ONE.

[88] Gavard J., Hou X., Qu Y., Masedunskas A., Martin D., Weigert R., Li X., Gutkind J.S. (2009). A role for a CXCR2/phosphatidylinositol 3-kinase gamma signaling axis in acute and chronic vascular permeability. Mol. Cell. Biol..

[89] Stamatovic S.M., Dimitrijevic O.B., Keep R.F., Andjelkovic A.V. (2006). Protein kinase Calpha-RhoA cross-talk in CCL2-induced alterations in brain endothelial permeability. J. Biol. Chem..

[90] Fletcher E.L., Dixon M.A., Mills S.A., Jobling A.I. (2023). Anomalies in neurovascular coupling during early diabetes: A review. Clin. Exp. Ophthalmol..

[91] Tang J., Kern T.S. (2011). Inflammation in diabetic retinopathy. Prog. Retin. Eye Res..

[92] Serra A.M., Waddell J., Manivannan A., Xu H., Cotter M., Forrester J.V. (2012). CD11b+ bone marrow-derived monocytes are the major leukocyte subset responsible for retinal capillary leukostasis in experimental diabetes in mouse and express high levels of CCR5 in the circulation. Am. J. Pathol..

[93] Ahn S.Y., Cho C.H., Park K.G., Lee H.J., Lee S., Park S.K., Lee I.K., Koh G.Y. (2004). Tumor necrosis factor-alpha induces fractalkine expression preferentially in arterial endothelial cells and mithramycin A suppresses TNF-alpha-induced fractalkine expression. Am. J. Pathol..

[94] Minami T., Aird W.C. (2005). Endothelial cell gene regulation. Trends Cardiovasc. Med..

[95] Greene J.A., Portillo J.A., Corcino Y.L., Subauste C.S. (2015). CD40-TRAF Signaling Upregulates CX3CL1 and TNF-α in Human Aortic Endothelial Cells but Not in Retinal Endothelial Cells. PLoS ONE.

[96] Asgeirsdóttir S.A., van Solingen C., Kurniati N.F., Zwiers P.J., Heeringa P., van Meurs M., Satchell S.C., Saleem M.A., Mathieson P.W., Banas B. (2012). MicroRNA-126 contributes to renal microvascular heterogeneity of VCAM-1 protein expression in acute inflammation. Am. J. Physiol. Renal. Physiol..

[97] Jobling A.I., Waugh M., Vessey K.A., Phipps J.A., Trogrlic L., Greferath U., Mills S.A., Tan Z.L., Ward M.M., Fletcher E.L. (2018). The Role of the Microglial Cx3cr1 Pathway in the Postnatal Maturation of Retinal Photoreceptors. J. Neurosci..

[98] Cardona A.E., Pioro E.P., Sasse M.E., Kostenko V., Cardona S.M., Dijkstra I.M., Huang D., Kidd G., Dombrowski S., Dutta R. (2006). Control of microglial neurotoxicity by the fractalkine receptor. Nat. Neurosci..

[99] Cardona S.M., Mendiola A.S., Yang Y.C., Adkins S.L., Torres V., Cardona A.E. (2015). Disruption of Fractalkine Signaling Leads to Microglial Activation and Neuronal Damage in the Diabetic Retina. ASN Neuro..

[100] Raoul W., Feumi C., Keller N., Lavalette S., Houssier M., Behar-Cohen F., Combadière C., Sennlaub F. (2008). Lipid-bloated subretinal microglial cells are at the origin of drusen appearance in CX3CR1-deficient mice. Ophthalmic. Res..

[101] Liu Z., Condello C., Schain A., Harb R., Grutzendler J. (2010). CX3CR1 in microglia regulates brain amyloid deposition through selective protofibrillar amyloid-β phagocytosis. J. Neurosci..

[102] Mendiola A.S., Garza R., Cardona S.M., Mythen S.A., Lira S.A., Akassoglou K., Cardona A.E. (2016). Fractalkine Signaling Attenuates Perivascular Clustering of Microglia and Fibrinogen Leakage during Systemic Inflammation in Mouse Models of Diabetic Retinopathy. Front. Cell. Neurosci..

[103] Church K.A., Rodriguez D., Vanegas D., Gutierrez I.L., Cardona S.M., Madrigal J.L.M., Kaur T., Cardona A.E. (2022). Models of microglia depletion and replenishment elicit protective effects to alleviate vascular and neuronal damage in the diabetic murine retina. J. Neuroinflamm..

[104] Vinet J., Weering H.R., Heinrich A., Kälin R.E., Wegner A., Brouwer N., Heppner F.L., Rooijen N., Boddeke H.W., Biber K. (2012). Neuroprotective function for ramified microglia in hippocampal excitotoxicity. J. Neuroinflamm..

[105] Bohlson S.S., O’Conner S.D., Hulsebus H.J., Ho M.M., Fraser D.A. (2014). Complement, c1q, and c1q-related molecules regulate macrophage polarization. Front. Immunol..

[106] Fonseca M.I., Chu S.H., Hernandez M.X., Fang M.J., Modarresi L., Selvan P., MacGregor G.R., Tenner A.J. (2017). Cell-specific deletion of C1qa identifies microglia as the dominant source of C1q in mouse brain. J Neuroinflamm..

[107] Morganti J.M., Nash K.R., Grimmig B.A., Ranjit S., Small B., Bickford P.C., Gemma C. (2012). The soluble isoform of CX3CL1 is necessary for neuroprotection in a mouse model of Parkinson’s disease. J. Neurosci..

[108] Lee S., Xu G., Jay T.R., Bhatta S., Kim K.W., Jung S., Landreth G.E., Ransohoff R.M., Lamb B.T. (2014). Opposing effects of membrane-anchored CX3CL1 on amyloid and tau pathologies via the p38 MAPK pathway. J. Neurosci..

[109] Cheung N., Mitchell P., Wong T.Y. (2010). Diabetic retinopathy. Lancet.

[110] Church K.A., Rodriguez D., Mendiola A.S., Vanegas D., Gutierrez I.L., Tamayo I., Amadu A., Velazquez P., Cardona S.M., Gyoneva S. (2023). Pharmacological depletion of microglia alleviates neuronal and vascular damage in the diabetic CX3CR1-WT retina but not in CX3CR1-KO or hCX3CR1(I249/M280)-expressing retina. Front. Immunol..

[111] Szukiewicz D. (2024). CX3CL1 (Fractalkine)-CX3CR1 Axis in Inflammation-Induced Angiogenesis and Tumorigenesis. Int. J. Mol. Sci..

[112] Jiang M., Xie H., Zhang C., Wang T., Tian H., Lu L., Xu J.Y., Xu G.T., Liu L., Zhang J. (2022). Enhancing fractalkine/CX3CR1 signalling pathway can reduce neuroinflammation by attenuating microglia activation in experimental diabetic retinopathy. J. Cell. Mol. Med..

[113] Sivandzade F., Prasad S., Bhalerao A., Cucullo L. (2019). NRF2 and NF-κB interplay in cerebrovascular and neurodegenerative disorders: Molecular mechanisms and possible therapeutic approaches. Redox. Biol..

[114] Cores Á., Piquero M., Villacampa M., León R., Menéndez J.C. (2020). NRF2 Regulation Processes as a Source of Potential Drug Targets against Neurodegenerative Diseases. Biomolecules.

[115] Lastres-Becker I., Innamorato N.G., Jaworski T., Rábano A., Kügler S., van Leuven F., Cuadrado A. (2014). Fractalkine activates NRF2/NFE2L2 and heme oxygenase 1 to restrain tauopathy-induced microgliosis. Brain.

[116] Rodriguez D., Church K.A., Smith C.T., Vanegas D., Cardona S.M., Muzzio I.A., Nash K.R., Cardona A.E. (2024). Therapeutic Delivery of Soluble Fractalkine Ameliorates Vascular Dysfunction in the Diabetic Retina. Int. J. Mol. Sci..

[117] Volin M.V., Woods J.M., Amin M.A., Connors M.A., Harlow L.A., Koch A.E. (2001). Fractalkine: A novel angiogenic chemokine in rheumatoid arthritis. Am. J. Pathol..

[118] Lee S.J., Namkoong S., Kim Y.M., Kim C.K., Lee H., Ha K.S., Chung H.T., Kwon Y.G., Kim Y.M. (2006). Fractalkine stimulates angiogenesis by activating the Raf-1/MEK/ERK- and PI3K/Akt/eNOS-dependent signal pathways. Am. J. Physiol. Heart Circ. Physiol..

[119] Ryu J., Lee C.W., Hong K.H., Shin J.A., Lim S.H., Park C.S., Shim J., Nam K.B., Choi K.J., Kim Y.H. (2008). Activation of fractalkine/CX3CR1 by vascular endothelial cells induces angiogenesis through VEGF-A/KDR and reverses hindlimb ischaemia. Cardiovasc. Res..

[120] Marchica V., Toscani D., Corcione A., Bolzoni M., Storti P., Vescovini R., Ferretti E., Palma B.D., Vicario E., Accardi F. (2019). Bone Marrow CX3CL1/Fractalkine is a New Player of the Pro-Angiogenic Microenvironment in Multiple Myeloma Patients. Cancers.

[121] El-Asrar A.M.A., Nawaz M.I., Ahmad A., de Zutter A., Siddiquei M.M., Blanter M., Allegaert E., Gikandi P.W., de Hertogh G., van Damme J. (2020). Evaluation of Proteoforms of the Transmembrane Chemokines CXCL16 and CX3CL1, Their Receptors, and Their Processing Metalloproteinases ADAM10 and ADAM17 in Proliferative Diabetic Retinopathy. Front. Immunol..

[122] Gough P.J., Garton K.J., Wille P.T., Rychlewski M., Dempsey P.J., Raines E.W. (2004). A disintegrin and metalloproteinase 10-mediated cleavage and shedding regulates the cell surface expression of CXC chemokine ligand 16. J. Immunol..

[123] Tsou C.L., Haskell C.A., Charo I.F. (2001). Tumor necrosis factor-alpha-converting enzyme mediates the inducible cleavage of fractalkine. J. Biol. Chem..

[124] Abel S., Hundhausen C., Mentlein R., Schulte A., Berkhout T.A., Broadway N., Hartmann D., Sedlacek R., Dietrich S., Muetze B. (2004). The transmembrane CXC-chemokine ligand 16 is induced by IFN-gamma and TNF-alpha and shed by the activity of the disintegrin-like metalloproteinase ADAM10. J. Immunol..

[125] Silva R., Malcangio M. (2021). Fractalkine/CX(3)CR(1) Pathway in Neuropathic Pain: An Update. Front. Pain. Res..

